# Nutritional and Microbiological Aspects of the Formulations and the Impact of Home Enteral Nutrition Therapy Use on Patients’ Quality of Life

**DOI:** 10.3390/medsci14010071

**Published:** 2026-02-04

**Authors:** Graciele Magda de Almeida, Mariana Buranelo Egea

**Affiliations:** 1 Faculty of Agronomy, Federal University of Goiás, Goiania 74690-900, GO, Brazil; gra.nutri@hotmail.com; 2 Goiano Federal Institute, Campus Rio Verde, Rio Verde 75906-900, GO, Brazil

**Keywords:** blenderized diets, food insecurity, feeding tube, food safety

## Abstract

**Background/Objectives**: Home Enteral Nutrition Therapy (HENT) is widely used for patients with preserved gastrointestinal function who cannot maintain adequate oral intake. It can be administered through commercial formulas (CF) or artisanal preparation (AP). **Methods**: This was a cross-sectional, descriptive, observational study with a quantitative and qualitative approach, conducted through semi-structured interviews by the researcher herself. Patients using HENT were evaluated for nutritional status using a 24 h dietary recall, and their quality of life was assessed using a questionnaire administered during an interview with the patient and/or caregiver. Microbial characteristics of the diets were evaluated by collecting samples and performing microbiological analyses according to standard methods. **Results**: 22 patients participated, mostly elderly, bedridden, and dependent, with gastrostomy as the primary method of administration (72.7%) and CF as the most commonly used (54.5%). AP consisted of cooked vegetables, legumes, milk, oil, and salt, and showed low nutritional diversity and a high risk of microbiological contamination due to manual handling. Frequent complications included diarrhea (72.7%) and mechanical complications (77.7%). Despite these issues, 91% of participants rated their quality of life as acceptable. **Conclusions**: HENT posed significant challenges to nutritional adequacy and microbiological safety, particularly among patients using artisanal preparations. These findings highlight the need for systematic monitoring and individualized adjustments by a multidisciplinary team, along with structured caregiver training, to optimize intake, reduce complications, and improve the quality and safety of home-based enteral therapy.

## 1. Introduction

Enteral Nutrition Therapy (ENT) is an alternative feeding method in which food is delivered in liquid form via a thin, soft, flexible plastic tube (enteral tube) directly to the stomach or small intestine [1,2]. This therapy is indicated when the patient cannot eat by mouth or cannot eat an adequate amount, based on the patient’s clinical and nutritional status, to supplement their oral intake, reduce the risk of infection and malnutrition, prevent intestinal mucosal atrophy, and prevent bacterial translocation [3].

This therapy can be used in hospitals and at home, where it is called home enteral nutrition therapy (HENT). HENT promotes the integration and rehabilitation of patients in their usual and familiar environments, improving psychological well-being and quality of life, reducing the risk of hospital infections, lowering costs associated with hospitalizations and readmissions, and increasing bed turnover in hospitals [4,5].

Most patients using HENT are often prescribed commercial formulas (CF). However, their use is usually unfeasible due to their high cost and the difficulty of obtaining them through subsidies or purchasing them with one’s own resources. In this case, homemade food-based preparations or blenderized formulations (which will be referred to in this text as artisanal preparations, AP) are indicated as an alternative, which has the advantage of using foods that may contain other compounds that are not present in CF, promoting an integrative environment regarding the patient’s and family’s nutrition, and maintaining the humanization of the treatment [6,7].

The literature is divided on whether to recommend CF or AP. However, it is essential to emphasize that although CF is more practical for daily use and medical prescriptions [3] AP can also be used. A nutritionist should monitor the use of AP to ensure that the entire diet meets the nutrient profile sufficient to meet these patients’ requirements and to guide them in reducing the risk of microbiological contamination [8]. AP can be considered more humanized and welcoming, as it fosters a relationship of affection and care between the patient and the caregiver/family member. However, they present some disadvantages in microbiological safety and nutritional needs [9]. Therefore, caregivers/family members must receive adequate guidance on preparation to reduce variations in diet composition, ensure adequate nutrient intake, and reduce the risk of contamination during preparation, handling, and administration of the diet, thereby avoiding potential complications in the patient’s health [10].

HENT is increasingly used to maintain nutritional status and prevent complications in patients who cannot meet their needs orally [11]. Beyond clinical benefits, however, HENT shifts some responsibility for care to families, often under conditions of limited resources and training [4]. In this context, the choice between CF and AP remains controversial. CF offer standardized composition and microbiological safety, but they may be costly or inaccessible in public systems [12]. AP, on the other hand, are culturally accepted and flexible, yet they may vary in nutrient content and pose a higher risk of contamination [6].

Despite increasing use of HEN, most studies examine isolated aspects such as cost, complications, or caregiver burden. Few have simultaneously evaluated nutritional adequacy, microbiological safety, and patient/caregiver experience in the home setting—particularly in low-resource contexts (as reviewed by our group [6]). Our study aims to address this gap by evaluating and describing patients’ and caregivers’ perceptions, experiences, and difficulties regarding attitudes and practices in HENT, as well as the nutritional composition and microbiological quality of the formulations and preparations used by these patients.

## 2. Materials and Methods

### 2.1. Study Protocol

This was a cross-sectional, descriptive, observational study with a quantitative and qualitative approach, conducted through semi-structured interviews by the researcher herself. The study was conducted in the municipality of Patrocínio (Minas Gerais, Brazil), with participants enrolled in the Nutritional Assistance Program (NAP). Data collection took place between September and November 2023, through home visits.

Interviews with patients and/or caregivers were conducted at their homes, with the researcher introducing themselves and explaining the research objectives and methods before obtaining consent. Eligible participants were adults (≥18 years) receiving HENT, residing within the municipality’s coverage area, registered in the program, and available for home visits. Unconscious patients could participate via a caregiver.

No sample size calculation was performed; the sample comprised all eligible patients during the study period. A total of 41 individuals (all participants in the NAP) were recruited. Of the 41 patients registered in the NAP, five died during the record collection period, six did not meet the inclusion criteria, five were not found at the registered addresses, and three had already removed the tube and progressed to an oral diet (Figure S1). The final sample consisted of 22 individuals divided into three groups according to the type of preparation used in the HENT, with the (i) AP group being fed an exlusively artisanal preparation of the food base (*n* = 06); (ii) AP + CF group being fed an artisanal preparation of the food + commercial formula (*n* = 04); and CF group being exclusively fed the commercial formula (*n* = 12).

### 2.2. Ethical Approval

The study was approved by the Ethics Committee of the Federal University of Goiás (CAAE: 68437923.9.0000.5083, 27 July 2023) and conducted in accordance with the Declaration of Helsinki. All participants (or their legal guardians) signed the Informed Consent Form.

### 2.3. Data Collection Instruments

#### 2.3.1. Participant Identification Questionnaire

Initially, sociodemographic and clinical data, along with information about its use at home, were collected to characterize the sample.

#### 2.3.2. Nutri Quality of Life Questionnaire

Patients’ quality of life was evaluated using a translated and adapted version of the Nutri Quality of Life Questionnaire (NutriQoL^®^) for the Brazilian population [13]. This questionnaire containing 34 positive and negative statements with three answer options was divided into (i) column A (17 statements) with positive sentences (from 1 to 9), where a score of −1 point was assigned to the answer “never”, 0 for “sometimes”, and 1 for “always”, and negative sentences (from 10 to 17), where a score of 1 was assigned to the answer “never”, 0 for “sometimes”, and −1 for “always”; and (ii) column B (17 statements) with positive and negative sentences, where a score of 1 was assigned to the answer “not at all important”, 2 for “moderately important”, and 3 for “very important”. Based on the sum of the scores, the quality of life of the patient receiving home enteral nutritional therapy was classified as very poor (0 to 20), bad (21 to 39), acceptable (40 to 60), good (61 to 80), and excellent (81 to 100). NutriQoL^®^ was verified for the entire study group and for stratified groups.

#### 2.3.3. Evaluation of the Nutritional Quality

The nutritional quality of the diets received by patients using food-based preparations or a combination of these with a CF (*n* = 10) was evaluated using a food frequency questionnaire, assessing weekly consumption (never, once, twice, three, four, five, six, or seven times a week) of certain food groups to prepare homemade preparations for patients undergoing HENT.

To assess the diet and estimate the nutrient and energy intake values of the diets, a 24 h recall was used, obtaining verbal information on food intake for the 24 h before the questionnaire, with data on the types of food and beverages used, including the method of preparation, and information on weight and/or portion size, in grams, milliliters, or household measures. Calorie and macronutrient intake (carbohydrates, proteins, and lipids) was calculated using only the Brazilian Food Composition Table (TBCA). To compare macronutrient intake, the Dietary Reference Intakes (DRIs) proposed by the Food and Nutrition Board (FND) and Resolution No. 21 [14] were used as references to assess variation in food consumption. Although different commercial brands were used, all CF prescribed in the CF group had the same caloric density (1.5 kcal/mL).

### 2.4. Evaluation of the Microbiological Safety of HENT Preparations

Microbiological quality was assessed in six home-prepared enteral nutrition samples. These samples were obtained through convenience sampling, based on caregiver availability and logistical feasibility during home visits. Of the six samples analyzed, four were from the AP group, and two were from the AP + CF group. No microbiological samples were collected from the exclusive CF group, as these products were industrially prepared and handled according to manufacturer instructions.

For each selected participant, a single sample (150 mL) was collected immediately before administration, with no repeated sampling. Samples were obtained directly from the diet container using disposable gloves and a graduated measuring device, following food safety procedures adapted from Food and Drug Administration (FDA) guidelines [15]. The samples were transferred to sterile, coded containers and transported in an isothermal box containing recyclable ice to maintain temperatures below 10 °C until arrival at the food microbiology laboratory for analysis.

The samples were subjected to research for *Mesophilic aerobes*, *Bacillus cereus*, *Coliforms*, *Escherichia coli*, *Listeria monocytogenes*, *Salmonella* sp., *Staphylococcus aureus*, *Yersinia enterocolitica*, and *Clostridium perfringens* according to methods recommended by the American Public Health Association (APHA), described in the Compendium of Methods for the Microbiological Examination of Foods [16]. The results were compared with the limits set in Resolution No. 503 [17], which defines the minimum requirements for Enteral Nutrition Therapy. Furthermore, comparisons with ESPEN, ASPEN, and FAO were made directly in the discussion item.

### 2.5. Statistical Analysis

Data were entered into a structured database and checked for consistency before analysis. Given the exploratory and descriptive nature of the study, no a priori sample size calculation was performed. Categorical variables are presented as absolute and relative frequencies. Continuous variables are summarized using means and standard errors, with minimum and maximum values provided.

Due to small and unequal group sizes (AP, *n* = 6; AP + CF, *n* = 4; CF, *n* = 12) and the non-normal distribution of the nutritional variables, non-parametric tests were used. Differences across the three preparation groups were explored using the Kruskal–Wallis test. When the overall test was statistically significant, pairwise post hoc comparisons were conducted using the Mann–Whitney U test with Bonferroni adjustment.

Exact *p*-values are reported. Effect sizes were calculated for pairwise comparisons using the rank-biserial correlation (r). Given the limited sample size and reduced statistical power, all inferential results should be interpreted as exploratory and hypothesis-generating rather than confirmatory. Statistical analyses were performed using STATISTICA, complemented by exact methods where appropriate.

## 3. Results

### 3.1. Sociodemographic and Clinical Characteristics of Patients Using HENT

Table 1 shows the distribution of individuals who used HENT in this study by sociodemographic characteristics. In the present study, females predominated in the age groups of 60 to 69 years (54.6%) and 70 to 79 years (18.2%), and in patients with only incomplete elementary education (41%). Meanwhile, patients’ marital status was nearly evenly split between single (36.4%) and married (31.8%).

Table 2 shows the distribution of clinical characteristics of individuals using HENT. A total of 72.7% of the individuals surveyed demonstrated neurological involvement (stroke, Alzheimer’s, and Parkinson’s diseases) as the initial disease that resulted in the need for ENT, followed by oncological diseases, and head trauma (13.6% each).

In the present study, gastrostomy (72.7%) was the most common access route, followed by the nasoenteral route (27.3%). None of the patients in the study used the jejunostomy route. 54.5% of the patients evaluated in the present study used the CF, followed by 27.3% who used an AP, and finally, 18.2% who used both. The CF reported were Nutren 1.0, Ensure, and Isousource 1.5.

Of the total patients evaluated, the present study’s sample could be stratified as described in Section 2.1.

### 3.2. Characteristics of HENT and Quality of Life of Patients

Table 3 shows the type of administration, number of administrations per day, and volume administered to the patients evaluated in the present study. A total of 50% of patients received the formulation by the gravitational method, followed by 34.6% by bolus. A total of 68.2% of the patients received 4 to 5 daily administrations, followed by 18.2% who received 2 to 3, and 13.6% received it 6 to 8. A total of 81.8% of the patients received 200–300 mL daily, followed by 100–200 mL and 400–500 mL (9% each). This study did not identify the type of continuous administration because it is more commonly used in hospitals.

Regarding complications during the use of HENT (Table 3), the patients evaluated in the present study reported the prevalence of mechanical complications such as displacement, accidental removal, obstruction and rupture of the tube (77.7%), diarrhea (72.7%), weight loss (68.6%), constipation (54.5%), and abdominal distension (41.4%). Aspiration pneumonia was reported in a smaller proportion of the sample (18.1%), and the present study did not observe nausea and vomiting.

About the sanitation of the vial, equipment, syringe, and other utensils, 100% of patients and/or caregivers reported performing it after each administration. The sanitation method of patients and/or caregivers was divided into 72.7% who used soap and water and 22.8% who reported rinsing with water only. In comparison, only 4.5% reported sanitizing with bleach/70% alcohol. Regarding the disposal of the vial, equipment, and/or syringe, 68.2% of patients and/or caregivers reported doing so daily, 18.2% weekly, and 13.6% every 2 days.

Finally, the quality-of-life questionnaire data (Table S1) were used to calculate patients’ quality-of-life scores. According to this score, 91% and 9% of patients on HENT have an acceptable and poor quality of life, respectively.

NutriQoL scores were similar across AP, AP + CF, and CF groups, with no clear between-group pattern. Values close to the overall average were demonstrated in the CF group (91.67% acceptable and 8.33% poor). On the other hand, all patients in the AP + CF group rated their quality of life as acceptable (100%), whereas 83.33% and 16.67% of patients in the AP group rated it as acceptable and poor, respectively. Caregivers completed most questionnaires due to patient dependency, which may have influenced their perceptions of quality of life. Furthermore, our sample size in each case is small; therefore, these results should be interpreted with caution.

### 3.3. Assessment of the Nutritional and Microbiological Qualities of Diets with AP Used in HENT

Nutritional (*n* = 10) and microbiological (*n* = 6) evaluations of the quality of the AP and AP + CF groups were conducted in this study. Table S2 shows the frequency of weekly consumption (never, once, twice, three, four, five, six, or seven times) of the foods used in artisanal preparations among HENT patients, as reported in the food frequency questionnaire.

Foods that were reported to be present in the diet every day were vegetables and salt (80%), beans and oil (70%), milk (60%), meat (50%), rice and pasta, and chicken (40%), eggs and fruits (30%), and greens (20%). Patients and/or caregivers reported never consuming/administering other legumes, seeds, cereals (chickpeas, lentils, and peas), artificial juice (powder, carton, concentrate, and soy), and fish and seafood.

Table 4 presents the results for the patient’s dietary intake volume, macronutrient content, and energy density obtained from the 24 h recall. Post hoc comparisons were restricted to predefined pairwise contrasts (AP vs. CF; AP vs. AP + CF; CF vs. AP + CF), with Bonferroni correction applied consistently across nutritional variables. Table S3 shows the *p*-values for comparisons between pairs of groups using the Mann–Whitney U test. Although some differences reached statistical significance, effect sizes ranged from moderate to large, supporting the practical relevance of nutritional disparities between preparation types, despite limited power. Although there was variation in the volume offered to the patients across the groups evaluated (1000.00–1500.00), there was no statistical difference between them. The highest energy density and energy values were found for the CF group (1.50 and 1637 kcal, respectively) (significantly different from AP and AP + CF groups), followed by the AP + CF (0.95 and 1220 kcal, respectively) and AP groups (0.88 and 967 kcal, respectively) (with no significant difference between them). The CF group presented the highest value of carbohydrates (229 g) (with a significant difference from the AP group) and lipids (46 g) (with a significant difference from the other groups). In comparison, the AP + CF group presented the highest value for protein content (74 g) (significantly similar to the CF group and different from the AP group).

Table 5 presents the results of the microbiological evaluation of the homemade preparations for all patients using HENT. All AP samples collected at home met the microbial standards for *Staphylococcus aureus*, *Salmonella* spp., *L. monocytogenes*, *Bacillus cereus*, *Clostridium*, and mesophilic aerobes, as required by Brazilian legislation. Regarding total coliforms, values above the limits permitted by Brazilian legislation were found in all samples collected. In contrast, for *Escherichia coli*, these values were observed in 33.3% of the collected samples.

## 4. Discussion

This study evaluated the quality of life and nutritional status of patients (*n* = 22) using HENT, as well as the microbiological quality of the diet. Only 18% of patients could complete the questionnaires without help from a caregiver or family member. The rest of the individuals studied did not have autonomy to perform activities of daily living, were bedridden, and/or non-verbal. This result is in line with other studies that have shown that patients using HENT were completely fragile and dependent on care, requiring full assistance from the caregiver and/or family member [10].

Although the sample in the present study was similar in terms of gender (male and/or female), the majority of individuals included in our cohort were in the elderly group (age > 60 years) (63.6%), who had only incomplete elementary education or were illiterate (59.2%) (Table 1). Other studies reported a mean age of 60 years [18], 68 years [19], or greater than 75 years [20], demonstrating a prevalence of elderly individuals using HENT. On the other hand, Van Aanholt, Matsuba, Dias, da Silva, and de Aguilar-Nascimento [7] demonstrated that among patients using HENT, individuals of all ages were represented, and only 20% were over 60 years old.

Most individuals included in the present study presented neurological disorders, represented by sequelae of stroke, Alzheimer’s, and Parkinson’s diseases (totaling 72.7%). This result agreed with other studies that demonstrated the prevalence of patients in HENT with neurological diseases (46.4% and 64% reported by Mazur et al. [21] and by Van Aanholt, Matsuba, Dias, da Silva and de Aguilar-Nascimento [7], respectively) on patients with stroke (31.2% reported by Menezes and Fortes [22]; dementia (26% reported by Menezes and Fortes [22]); and oncological patients (22.5% and 18% reported by Menezes and Fortes [22] and Van Aanholt, Matsuba, Dias, da Silva and de Aguilar-Nascimento [7], respectively).

In line with other studies [7,19,21,22], gastrostomy was the route of administration in our cohort. Gastrostomy feeding is indicated when long-term enteral nutrition is required (>4 weeks), whereas nasogastric tube feeding is preferred for short-term use [10]. Although gastrostomy is an invasive procedure and may lead to complications such as wound infection or leakage [23], it is often favored because it allows use of the gastrointestinal tract, improving tolerance and supporting metabolic and nutritional status [24].

The present study demonstrated that more than 50% of the individuals interviewed used AP or a combination of AP and CF. In other regions of the country, this number may vary, for example, to ~5% in Brasília (Federal District, Brazil) [22] and ~72% in Curitiba (Paraná, Brazil) [21]. In Brazil, CF can be provided in home care, depending on the assessment, ranging from 50% to 100% of the patient’s energy needs [6]. Commercial formulas are considered a major challenge by managers at all three levels of Brazil’s Unified Health System (SUS) due to their high cost and divergent interpretations of governmental policies, which contribute to unequal allocation of financial resources across regions of the country [25]. This is in line with Wong, Goh, Banks and Bauer [18] demonstrated in their study. These authors demonstrated that lower-middle-income Asian countries in the Pacific region use preparations based on AP (40%) and AP + CF (60%).

The literature has highlighted the advantages of using CF, including knowledge of the nutrient levels it contains, stability of the nutrients and their physical-chemical characteristics, ease of handling with specialized guidance, and microbiological safety [6,26]. However, in our cohort, reliance on CF appears closely linked to food insecurity among patients receiving HENT (Table S1), as food insecurity spans from concern about food availability to extreme hunger [27]. Most patients and/or caregivers reported uncertainty about continued access to CF through government assistance programs (86.5%), which caused distress and insecurity about sustaining adequate home nutritional care.

In contrast to findings reported in the literature [6,28], many respondents (63.6%) indicated that artisanal preparations did not meet their preferences for sensory characteristics such as texture, color, aroma, temperature, and taste. This finding highlights a tension between the cultural and symbolic value of AP and its practical limitations in meeting individual sensory expectations in home enteral nutrition. However, the AP, when carefully planned, can be innovative, offering not only the necessary nutrition but also contributing to a more profound sense of identity, belonging, and humanization linked to the issue of behavior and eating experience, which can result in improvements in the physical and emotional health of the individual on ENT [29].

Although one advantage of AP is maintaining a variety of foods in the diet, in the present study, patients and/or caregivers reported including only beef, milk, vegetables, beans, cooking oil, and salt (Table S2) because they believed the patient was already adapted and would be insecure about diversifying, which could result in complications or intolerance. These ingredients were prepared by boiling them in water (except for the fruit mixture) and then homogenized.

These AP preparation steps, trituration and homogenization, require the addition of more liquids to reduce and remove particles that could cause tube clogging [30,31]. This may indicate that any dietary survey used to measure the nutritional values administered by HENT may have been overestimated in our cohort (Table 4), and, therefore, this data should be interpreted with caution. There is a lack of standardization in food preparation procedures in AP (cooking time, amount of water, sieving, amount of retained residues, and types of mixtures), which has been associated with variation in composition and nutritional inadequacy of these diets [6].

For this reason, regular nutritional monitoring and the use of tools that help standardize the preparation of this type of diet are important [32]. As with oral nutrition, it is essential that the stages of food preparation do not significantly alter nutrient content and that they provide adequate nutrition to the patient, ensuring a balanced composition of macro- and micronutrients. The nutritionist must determine nutritional requirements and the adequacy of home-prepared foods, and identify and guide appropriate techniques for handling these foods for ENT [33].

The results obtained in the present study regarding energy value and macronutrient content appeared to be inconsistent across the AP, AP + CF, and CF groups. Regarding energy density, AP was classified as a low-energy-density formula, AP + CF as a normal-energy-density formula, and CF as a high-energy-density formula [14]. The energy value of CF is related to the higher lipid content, while the energy value of the AP + CF group is associated with the carbohydrate content. Interestingly, protein content increased when CF and AP were used together, making it more suitable for bedridden people with significant muscle loss due to inactivity.

Furthermore, because most patients were highly dependent, dietary information was provided primarily by caregivers rather than by the patients themselves. Caregiver reports often reflect the prescribed or planned enteral volume rather than the amount actually administered and retained, and they often overlook sources of loss such as regurgitation, vomiting, feeding interruptions, leakage, and tube residues—events that are rarely perceived or systematically recorded. Consequently, calculated nutrient intake may overestimate actual net intake, particularly among patients experiencing complications or feeding intolerance.

The fact that AP was classified as a low-energy-density food and that CF is either financially inaccessible or inconsistently supplied reinforces the importance of the multidisciplinary team. Dietitians can modify homemade recipes to boost energy density by adding oils, powdered milk, nut pastes, or starches, while maintaining the necessary viscosity for tube feeding [34]. Nurses can advise caregivers on infusion schedules that facilitate more frequent or evenly spaced feedings, and physicians can monitor tolerance and adjust prescriptions as needed [35]. When carefully planned and supervised, AP can be nutritionally optimized, but ongoing monitoring remains crucial, and therefore, these data may reflect a specific issue.

In the present study, the lack of multidisciplinary monitoring may have led to a negative energy balance, resulting in weight loss or weight maintenance, as reported by 31.8% who indicated that the patient never regained the weight. Weight loss or failure to regain weight may reflect a combination of insufficient energy intake, disease-related catabolism, and limited functional mobility [36].

In the present study, although administered volumes were generally adequate, the number of daily feedings was lower than recommended, which may have contributed to reported discomfort and complications. Volume tolerance and the method of dietary administration should be considered when developing a food preparation, as it may be challenging to meet the nutritional needs of patients sensitive to increased HENT volume, and rapid administration may cause diarrhea and other complications [8,29].

Another issue that may lead to complications, such as diarrhea, could be related to equipment sanitation and the microbiological quality parameters that must also be maintained for HENT. An interesting finding from our study is that, although all patients and/or caregivers reported cleaning bottles, equipment, syringes, and other utensils after each use—most using only soap and water—microbiological contamination was still found in several samples. This discrepancy suggests that the problem may not be a lack of awareness of hygiene recommendations but rather a failure to properly execute sanitation procedures. In home settings, caregivers often rely on improvised materials, incorrect disinfectant concentrations, insufficient contact times, or inappropriate drying and storage conditions. These observations highlight a gap between “knowing” and “doing,” emphasizing that written guidance alone is not enough. Effective contamination prevention likely requires structured, practical, and repeated training, including demonstrations, supervision, and feedback. In this way, the caregiver or family member preparing the formulations must receive proper guidance to minimize variations in composition, ensure sufficient nutrient supply, and reduce the risk of contamination during preparation, handling, and administration—thus helping to prevent potential health complications [6].

Furthermore, as with foods prepared for oral therapy, microbiological quality standards must be maintained when preparing HENT. As evidenced in this study, AP made from blended foods was contaminated with total coliforms, an indicator of sanitary quality, and *Escherichia coli*. This contamination can occur due to inadequate handling; improper sanitation of physical facilities, equipment, and utensils; deficient aseptic techniques; inadequate storage and refrigeration of formulations; and poor water quality used in the preparation of artisanal preparations and in sanitizing equipment and utensils [11,37,38]. This highlights the need to train individuals involved in the formulation production process.

On the other hand, the ESPEN Practical Guideline on Home Enteral Nutrition and ASPEN, although they provide guidance on safety, preparation, handling, and monitoring practices for enteral nutrition to reduce risks, do not specify values for comparison with results [39,40]. However, for food products, FDA guidelines suggest that aerobic counts greater than 10^4^ CFU/g in a single sample or coliform counts > 3 organisms/g are generally considered unacceptable for consumption, and the presence of pathogens such as *Salmonella* or *Listeria* is prohibited—standards that are analogous to acceptable practice for enteral feeds [41]. These comparisons suggest that the microbiological thresholds used in this study are compatible with internationally recognized safety principles, strengthening the relevance of our findings beyond the local context. Therefore, hygiene and sanitary requirements are applied in the handling, storage, and administration stages of these preparations to ensure their safety [42].

Because microbiological sampling was performed at a single time point and under heterogeneous household conditions, including variations in food handling, storage practices, water sources, and ambient temperatures, the findings should be interpreted as context-specific. They may not represent the full range of contamination risks associated with home-prepared enteral nutrition.

Although the rates of complications such as tube obstruction/rupture and diarrhea were high in our cohort, the majority of respondents nonetheless rated their quality of life as “acceptable”. Quality-of-life scores were broadly similar across AP, AP + CF, and CF groups, suggesting that perceived well-being may be influenced more by contextual and psychosocial factors than by preparation type alone.

This apparent paradox may reflect several factors. First, quality of life is a subjective construct—patients and caregivers may prioritize the benefits of remaining at home, autonomy, and the ability to maintain oral contact with family over the discomfort associated with complications [43]. Second, individuals and caregivers often adapt to chronic interventions over time, such that frequent but manageable complications may be perceived as part of the routine rather than overwhelming burdens [44]. Third, the instrument used to assess quality of life may emphasize broader domains (functional, emotional, social) that are not directly captured by isolated clinical events [45]. Finally, the lack of alternative (e.g., prolonged hospitalization) might lead participants to evaluate their overall experience more positively despite frequent complications [46]. These factors could help reconcile the high complication rates with predominantly acceptable quality-of-life ratings.

Many family members report that moving to the home is positive, allowing them to be closer to their family members. However, caregivers may experience conflicting feelings when faced with tasks they have never performed before that require physical, psychological, social, intellectual, and financial resources they often lack [47]. In general, patients and/or caregivers in the present study reported feeling unprepared, without training or education and support from health professionals, without even knowing how to prepare enteral nutrition, the correct position for feeding, and feeding times, thus leading to errors and resulting in negative consequences for the patient and family, as previously reported by Afonso, Arroyo, Gastaldi, Assalin, Yamamura and Girão [4]. Thus, it seems evident that patients and/or caregivers lack information about their condition and its daily treatment, as well as adequate food.

It seems possible to develop nutritionally and microbiologically appropriate AP through qualified professional monitoring and adequate guidance on the use of homemade measures and preparation methods, in addition to good dietary management practices, thus allowing caregivers to have the freedom to choose a variety of foods while emphasizing the importance of the nutritionist as a qualified professional in developing a meal plan with adequate amounts of nutrients [48,49]. Thus, it is possible to ensure that the patient and/or caregiver has the opportunity to choose their food, thereby reducing food insecurity.

Although this study addressed a topic that is rarely explored and often disregarded in the literature, it has several limitations. First, the sample was relatively small, drawn from a single municipality, and recruited for convenience, which limits the generalizability of the findings. The small and unequal group sizes substantially limited the statistical power of this study and constrained the robustness of between-group comparisons. Accordingly, the analyses were conducted with an exploratory and descriptive intent, and the findings should be interpreted with caution rather than as definitive evidence of group differences. Nonparametric methods were used to minimize distributional assumptions, and multiple-testing corrections were applied to reduce the risk of type I error. Nevertheless, limited power increases the likelihood of type II error; therefore, non-significant results should not be interpreted as evidence of equivalence between groups, and statistically significant findings should be regarded as hypothesis-generating signals that require confirmation in larger studies.

Second, a large number of patients using HENT were unable to answer the quality-of-life questionnaire because they were unconscious due to their underlying disease, introducing the potential for recall and reporting bias. Third, dietary intake was assessed using a single 24 h recall, which may not reflect habitual consumption. Furthermore, the nutritional values reported in this study may be overestimated because dietary surveys do not account for food residues retained during the sieving process required for tube administration. Furthermore, limitations in microbiological sampling have been discussed previously.

Taken together, these results demonstrate that many caregivers receive fragmented information from a wide variety of healthcare professionals and often have to make decisions that affect patients’ quality of life. This reinforces the need for educational strategies that include structured training sessions, step-by-step demonstrations, written and visual materials adapted to literacy levels, and periodic reinforcement visits. Regular follow-up by a multidisciplinary team—particularly dietitians—is essential to adjust nutrient prescriptions, monitor tolerance, and prevent complications. Simple checklists and home-based protocols can improve microbiological safety and reduce tube obstruction and diarrhea.

Policies that facilitate equitable access to commercial formulas and support the safe preparation of blenderized diets when indicated may minimize food insecurity and improve quality of life in home enteral nutrition. All these practical implications may seem simple, but they ultimately pose a challenge for most countries.

## 5. Conclusions

This study identified a profile of users of HENT characterized by an elderly and clinically fragile population, predominantly affected by neurological diseases, who commonly rely on gastrostomy feeding and commercial formulas. Although commercial formulas were perceived as safer from a technical and hospital care perspective, patients and caregivers expressed uncertainty about their suitability for home use regarding nutrition, social support, and cost.

Regarding the HENT experience, many caregivers reported that transitioning care to the home environment was perceived positively because it increased proximity to family members. However, this transition also imposed new and demanding responsibilities that require physical, psychological, social, and financial resources, often beyond caregivers’ capacity, and substantially affected daily family life.

Our findings indicate that artisanal preparations frequently present nutritional and microbiological limitations. Nevertheless, these findings do not preclude their use. When adequately planned, handled under appropriate hygiene conditions, and supported by professional guidance, artisanal preparations may represent a viable and culturally meaningful alternative for home enteral feeding.

These results underscore the importance of structured education, practical training, and continuous monitoring by a multidisciplinary healthcare team to ensure nutritional adequacy and microbiological safety without compromising patient health. Future studies with larger samples and longitudinal designs are warranted to confirm these findings and support the development of evidence-based guidelines for home enteral nutrition.

## Figures and Tables

**Table 1:** Distribution of individuals using home enteral nutrition therapy according to gender, age, marital status, and education status of patients using home enteral nutrition therapy (%) (*n* = 22).

Variables	Percentage (%)
Gender	
Male	45.4
Female	54.6
Age (years)	
18–29	13.7
30–39	13.7
40–49	-
50–59	9.0
60–69	18.2
70–79	18.2
80–89	9.0
90+	18.2
Marital Status	
Married	31.8
Widower	31.8
Single	36.4
Education Status	
Illiterate	18.2
An incomplete elementary school	41.0
Complete elementary school	9.0
Incomplete high school	4.5
Complete high school	27.3

**Table 2:** Underlying disease, time of use of enteral diet, route of diet administration, and type of diet proposed for enteral diet of patients using home enteral nutrition therapy (%) (*n* = 22).

Variables	Percentage (%)
Underlying disease	
Neurological (Alzheimer’s, Parkinson’s, and stroke)	72.7
Oncological	13.6
Head trauma	13.6
Time of use of the enteral diet (months)	
<06	9.0
6–12	31.9
12–24	9.0
24–60	27.3
60–120	4.5
>120	18.2
Route of diet administration	
Nasoenteral	27.3
Gastrostomy	72.7
Type of diet proposed	
Commercial formula	54.5
Artisanal preparation	27.3
Artisanal preparation + commercial formula	18.2

**Table 3:** Type of administration, number of administrations per day, volume administered, and complications of patients using home enteral nutrition therapy (%) (*n* = 22).

Variables	Percentage (%)
**Type of administration**	
Gravitational	50.0
Bolus	36.4
Both	13.6
**Number of administrations per day (times)**	
2 to 3	18.2
3 to 5	68.2
6 to 8	13.6
**Volume administered (mL)**	
100 to 200	9.0
200 to 300	81.8
300 to 400	-
400 to 500	9.0
**ENT complications**	
Diarrhea	72.7
Abdominal distension	41.4
Aspiration pneumonia	18.1
Nausea/vomiting	-
Constipation	54.5
Mechanical complications	77.7
Weigh loss	68.6

**Table 4:** The volume of food offered, energy density, and nutrient content of daily intake of patients using home enteral nutrition therapy being (i) AP group with individuals feed exclusiveness artisanal preparation of the food base (*n* = 06); (ii) AP + CF group with individuals feed with artisanal preparation with food + commercial formula (*n* = 04); and CF group with individuals feed with exclusive commercial formula (*n* = 12).

Group		Offered Volume (mL)	Energy Density	Energy (kcal)	Carbohydrates (g)	% Diet	Proteins (g)	% Diet	Lipids (g)	% Diet
AP	Mean ± SE	1100.00 ± 109.54	0.88 ± 0.08	967.00 ± 133.39	165.00 ± 26.17	68.25	40.50 ± 7.71	16.75	16.33 ± 7.17	15.19
	Low–High	1000.00–1200.00	0.74–0.99	744.00–1100.00	122.00–192.00		28.00–47.00		9.00–30.00	
AP + CF	Mean ± SE	1300.00 ± 244.94	0.95 ± 0.14	1220.00 ± 175.99	179.50 ± 29.35	58.85	74.00 ± 27.91	24.26	26.00 ± 7.43	19.18
	Low–High	1000.00–1500.00	0.84–1.16	1005.00–1400.00	155.00–211.00		45.00–112.00		21.00–37.00	
CF	Mean ± SE	1092.00 ± 156.42	1.50 ± 0.00	1637.00 ± 234.64	229.00 ± 32.84	55.95	64.00 ± 19.89	15.63	46.00 ± 13.90	25.29
	Low–High	1000.00–1500.00	1.50–1.50	1500.00–2250.00	210.00–315.00		63.00–94.50		45.00–67.00	
H	-	3.2427	18.7529	17.3128	12.9407	15.0596	12.1533	12.9806	17.3231	18.9868
*p*	-	0.1976	0.0001	0.0002	0.0015	0.0005	0.0023	0.0015	0.0002	0.0001

H: Kruskal–Wallis test statistic. A *p*-value < 0.05 indicates a significant difference between treatments according to the Kruskal–Wallis test. % diet: The percentage contribution of that macronutrient (left column) to the total caloric value of the diet. All commercial formulas used in the CF group had an energy density of 1.5 kcal/mL; therefore, no variability was observed within this group. Concerning the distribution of macronutrients in the diet, carbohydrates ranged from 55–69%, proteins from 15–25%, and lipids from 15–26%. The highest values were found for carbohydrates in the AP group (significantly different from the CF group, *p* < 0.00), proteins in the AP + CF group (significantly different from the other groups), and lipids in the CF group (significantly different from the other groups).

**Table 5:** Microbiological evaluation of artisanal preparations used by patients in Home Enteral Nutritional Therapy (*n* = 6).

Microorganisms (CFU/mL) *	BL	Sample 1	Sample 2	Sample 3	Sample 4	Sample 5	Sample 6
Aerobic mesophiles	<10^3^	158	200	190	200	101	108
*Bacillus cereus*	<10^3^	<3	<3	<3	<3	<3	<3
Coliforms	<3	1.100	2.400	1.100	1.700	104	380
*Escherichia coli*	<3	0	8	9	0	0	0
*Listeria monocytogenes*	Absent	Absent	Absent	Absent	Absent	Absent	Absent
*Salmonella* sp.	Absent	Absent	Absent	Absent	Absent	Absent	Absent
*Staphylococcus aureus*	<3	<3	<3	<3	<3	<3	<3
*Yersinia enterocolitica*	Absent	Absent	Absent	Absent	Absent	Absent	Absent
*Clostridium perfrigens*	<10^3^	<3	<3	<3	<3	<3	<3

* For comparison, Brazilian legislation indicates that for liquids, the density should be considered equal to 1 and mL treated as g. BL: Brazilian legislation. Samples 1, 2, 3, and 4 from the AP group and Samples 5 and 6 from the AP + CF group. CFU: colony-forming unit.

## Data Availability

The original contributions presented in this study are included in the article/Supplementary Material. Further inquiries can be directed to the corresponding author.

## Supplementary Materials

The following supporting information can be downloaded at: https://www.mdpi.com/article/10.3390/medsci14010071/s1, Figure S1: Schematic of the distribution of individuals using home enteral therapy who were recruited, selected, and grouped in the present study; Table S1: Quality of life questionnaire carried out with patients in Home Enteral Nutritional Therapy (HENT); Table S2: Frequency (%) of weekly consumption of foods used in artisanal preparations or artisanal preparation combined with commercial formula of patients using home enteral nutrition therapy obtained by the food frequency questionnaire (*n* = 10); Table S3. *p*-values for comparisons between pairs of groups using the Mann-Whitney U test
