# Supplementary material for: Nutritional and Microbiological Aspects of the Formulations and the Impact of Home Enteral Nutrition Therapy Use on Patients’ Quality of Life
Source: Med Sci (Basel). 2026 Feb 4;14(1):71. doi: 10.3390/medsci14010071 (PMC12922074; doi:10.3390/medsci14010071)
Supplement: Supplementary file 1 [file medsci-14-00071-s001.zip › medsci-4119636-supplementary.pdf]

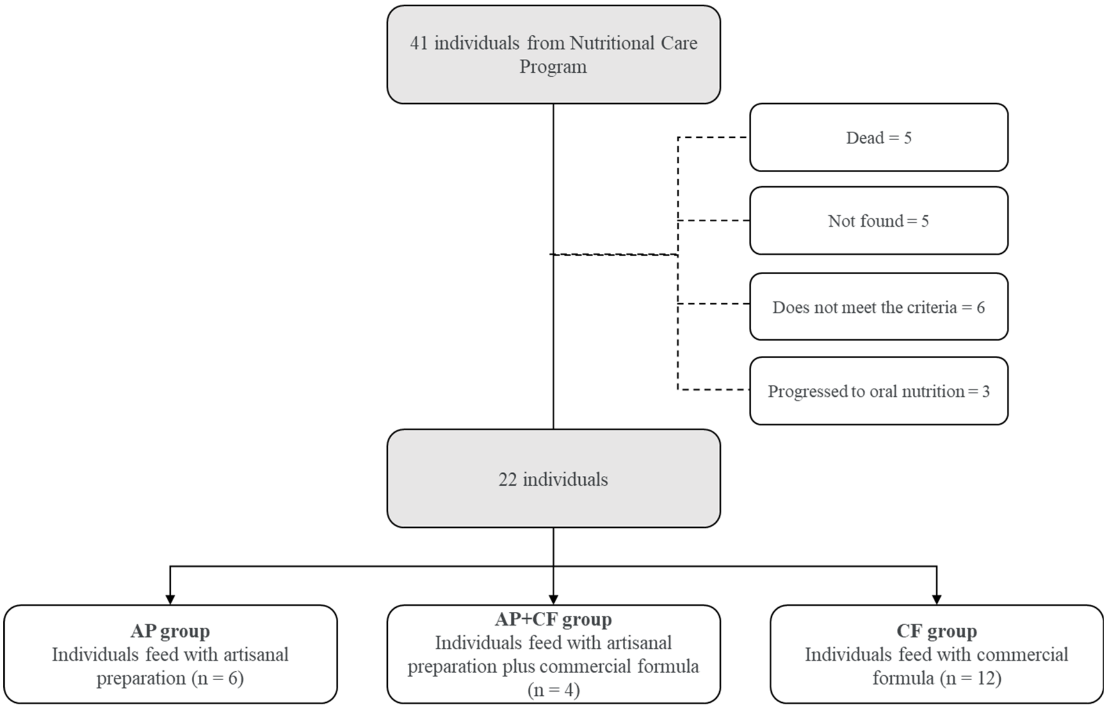

Figure S1. Schematic of the distribution of individuals using home enteral therapy who were recruited, selected, and grouped in the present study

Table S1. Quality of life questionnaire carried out with patients in Home Enteral Nutritional Therapy (HENT)

Question	Total	Percentage (%)
**1 - With HENT, I maintain my usual eating times (example: breakfast, lunch, snack, and dinner):**		
Never	5	22.73
Sometimes	15	68.18
Always	2	9.09
**2 - Keeping my regular eating times, for me, it means:**		
Nothing important	0	0.00
Moderately important	4	18.18
Very important	18	81.82
**3 - HENT adjusts to my preferences for food characteristics (example: texture, color, smell, temperature, and taste):**		
Never	14	63.64
Sometimes	6	27.27
Always	2	9.09
**4 - HENT adjusts to my preferences for food characteristics, for me, it is:**		
Nothing important	1	4.55
Moderately important	5	22.73
Very important	16	72.73
**5 - Since taking HENT, I move more easily and feel more agile:**		
Never	17	77.27
Sometimes	4	18.18
Always	1	4.55
**6 - Being able to move more easily, feeling more agile, for me is:**		
Nothing important	0	0.00
Moderately important	1	4.55
Very important	21	95.45
**7 - With HENT I can continue doing my daily activities (e.g., reading the newspaper, cooking, washing the car, cleaning, watching TV):**		
Never	18	81.82
Sometimes	4	18.18
Always	0	0.00

**8 - Being able to continue doing my daily activities, for me, is:**		
Nothing important	0	0.00
Moderately important	1	4.55
Very important	21	95.45
**9 - Since taking HENT, I see that my physical appearance is improving (example: I see myself healthier):**		
Never	9	40.91
Sometimes	13	59.09
Always	0	0.00
**10 - My physical appearance is improving, for me it is:**		
Nothing important	0	0.00
Moderately important	3	13.64
Very important	19	86.36
**11 - Obtaining HENT preparations is simple (e.g., I buy them online, I buy them in a physical store, they are provided by the municipality) or I follow the homemade recipe:**		
Never	2	9.09
Sometimes	17	77.27
Always	3	13.64
**12 - Getting or easily executing HENT preparations, for me, is:**		
Nothing important	0	0.00
Moderately important	0	0.00
Very important	22	100.00
**13 - With HENT, I am confident that I am well nourished:**		
Never	2	9.09
Sometimes	11	50.00
Always	9	40.91
**14 - Trusting that I am well nourished, for me, is:**		
Nothing important	0	0.00
Moderately important	0	0.00
Very important	22	100.00
**15 - With HENT, I regained weight:**		
Never	7	31.82
Sometimes	10	45.45
Always	5	22.73
**16 - Regaining weight, for me, is:**		
Nothing important	0	0.00
Moderately important	0	0.00

Very important	22	100.00
**17 - HENT allows me to go out with my friends and/or family:**		
Never	17	77.27
Sometimes	4	18.18
Always	1	4.55
**18 - Being able to go out with my friends and/or family, for me, is:**		
Nothing important	0	0.00
Moderately important	2	9.09
Very important	20	90.91
**19 - Using the probe for ENT harms my skin (e.g., dryness, irritation, infections):**		
Never	5	22.73
Sometimes	15	68.18
Always	2	9.09
**20 - That my skin is harmed by using the probe, for me it is:**		
Nothing important	0	0.00
Moderately important	1	4.55
Very important	21	95.45
**21- HENT prevents me from sleeping well:**		
Never	3	13.64
Sometimes	15	68.18
Always	4	18.18
**22 - Sleeping well, for me, is:**		
Nothing important	0	0.00
Moderately important	0	0.00
Very important	22	100.00
**23 - I worry that my body will get used to HENT, and I won't be able to go back to eating like before:**		
Never	8	36.36
Sometimes	10	45.45
Always	4	18.18
**24 - My body is getting used to HENT, and I can't go back to eating like before, for me it's:**		
Nothing important	0	0.00
Moderately important	1	4.55
Very important	20	90.91
**25 - With HENT, I miss chewing and tasting food:**		
Never	3	13.64
Sometimes	9	40.91

Always	10	45.45
**26 - Chewing and tasting food, for me, is:**		
Nothing important	0	0.00
Moderately important	0	0.00
Very important	22	100.00
**27 - With HENT, I feel physical discomfort due to eating (e.g., heavy stomach, heartburn, dry mouth, and reflux):**		
Never	10	45.45
Sometimes	9	40.91
Always	3	13.64
**28 - Feeling physical discomfort due to food, for me, is:**		
Nothing important	0	0.00
Moderately important	0	0.00
Very important	22	100.00
**29 - With HENT, my family and/or caregiver can better monitor my diet:**		
Never	0	0.00
Sometimes	10	45.45
Always	12	54.55
**30 - My family and/or caregiver should better monitor my diet, for me it is:**		
Nothing important	0	0.00
Moderately important	1	4.55
Very important	21	95.45
**31- With HENT, I limit activities with my friends to activities that do not involve food:**		
Never	0	0.00
Sometimes	1	4.55
Always	21	95.45
**32 - Limiting activities with my friends to activities that don't involve food, for me, is:**		
Nothing important	0	0.00
Moderately important	1	4.55
Very important	21	95.45
**33 - Since taking HENT, I am more concerned about my health:**		
Never	1	4.55
Sometimes	1	4.55
Always	20	90.91
**34 - Being more concerned about my health, for me, is:**		
Nothing important	0	0.00

Moderately important	0	0.00
Very important	22	100.00

Table S2. Frequency (%) of weekly consumption of foods used in artisanal preparations or artisanal preparation combined with commercial formula of patients using home enteral nutrition therapy obtained by the food frequency questionnaire (n=10)

**Food types**	**Number of days per week**							
	**7**	**6**	**5**	**4**	**3**	**2**	**1**	**Never**
Greens (lettuce, arugula, watercress, kale, and spinach)	20				10		30	40
Vegetables (potatoes, cassava, yams, taro, carrots, and beets)	80			10		10		
Fruits	30	10				10	20	30
Beans	70					10	10	10
Other legumes, seeds, and cereals (chickpeas, lentils, and peas)								100
Rice and pasta	40		10	20		10		20
Meat	50	10	20				10	10
Chicken	40	10	10	20				20
Fish and seafood								100
Eggs	30	20	10			20	10	10
Milk	60	10			10	10		10
Oil	70							30
Olive oil						10		90
Sugar, honey, and brown sugar				10				90
Salt	80							20
Artificial juice (powder, carton,								100

concentrate, and soy)

Table S3. p-values for comparisons between pairs of groups using the Mann-Whitney U test

Comparations	p-value								
	**Offered volume (mL)**	**Energy density**	**Energy (kcal)**	**Carbohydrates (g)**	**% diet**	**Proteins (g)**	**% diet**	**Lipids (g)**	**% diet**
AP vs. CF	0.681965	0.000108	0.000108	0.000108	0.000108	0.000108	0.290778	0.000108	0.000108
AP vs AP+CF	0.257143	0.609524	0.066667	0.609524	0.066667	0.038095	0.019048	0.066667	0.352381
CF vs AP+CF	0.132967	0.001099	0.001099	0.058242	0.170330	0.683516	0.001099	0.001099	0.001099

% diet: The percentage contribution of that macronutrient (left column) to the total caloric value of the diet. Effect sizes (r) for Mann–Whitney comparisons are provided to support interpretation of the magnitude of observed differences, independent of statistical significance.
